# The value of fecal calprotectin in *Clostridioides difficile* infection: A systematic review

**DOI:** 10.3389/fphys.2022.881816

**Published:** 2022-08-03

**Authors:** Bao-Jiang Wen, Li-Ger Te, Xiao-Xuan Liu, Jian-Hong Zhao

**Affiliations:** ^1^ The Second Hospital of Hebei Medical University, Shijiazhuang, Hebei, China; ^2^ Hebei Provincial Center for Clinical Laboratories, Shijiazhuang, Hebei, China; ^3^ Graduate School of Hebei Medical University, Shijiazhuang, Hebei, China

**Keywords:** *Clostridioides difficile* infection, fecal calprotectin, biomarker, value, systematic review

## Abstract

As a marker of inflammation, calprotectin has potential application value in a variety of inflammatory diseases, such as arthritis and bacterial infections. *Clostridioides difficile* infection (CDI) is an infectious disease that causes intestinal damage and inflammation. This systematic review aims to determine whether fecal calprotectin has application value in CDI. Nine databases were searched from inception to 6 June 2022, and 17 studies were included. These studies were divided into four groups according to their content. Generally speaking, fecal calprotectin is not an ideal indicator for the diagnosis and prognosis prediction of CDI but may serve as a potential indicator for assessing disease severity and as a readily detectable marker for CDI screening. In addition, patients in need of treatment or with detectable toxins in stool may tend to have higher levels of fecal calprotectin. In summary, fecal calprotectin has some potential application value in CDI. However, further studies are needed to verify these findings and determine the reliability of calprotectin as a biomarker for CDI.

## 1 Introduction

*Clostridioides difficile* is an anaerobic, spore-forming, Gram-positive bacterium that is considered to be the main cause of antibiotic-associated diarrhea (AAD) and healthcare-associated infections (Khanna and Pardi, 2012). Various clinical manifestations have been reported for *C. difficile* infection (CDI) from asymptomatic colonization to mild and self-limiting diarrhea to severe fulminant colitis characterized by hypotension, shock, megacolon or intestinal obstruction (McDonald et al., 2018). In the United States, CDI affected 224,000 people and caused approximately 13,000 deaths in 2017 alone, with medical costs estimated at $1 billion (Guh et al., 2020). Therefore, the accurate diagnosis and prevention of CDI are of high importance.

CDI is characterized by three unformed stools in 24 h and the confirmation of the presence of toxigenic *C. difficile* through laboratory testing (McDonald et al., 2018). Currently, commonly used laboratory assays for diagnosing CDI include toxin-producing cultures, glutamate dehydrogenase (GDH), nucleic acid amplification assays (NAAT) and toxin A/B enzyme immunoassays (EIA) (Lee et al., 2021). Although easy to use and affordable, these tests have limitations. In particular, because the results can only qualitatively indicate the existence of GDH and toxin A/B but cannot provide quantitative measurements, they cannot be used to judge the severity of CDIs (Hassanain et al., 2021). In addition, a positive *C. difficile* test does not always indicate a clinical infection that requires treatment. The fact that the asymptomatic colonization rate of *C. difficile* is 3.4–8.1% upon admission further challenges the diagnosis of CDI (Zacharioudakis et al., 2015; Longtin et al., 2016; Meltzer et al., 2019). In a single-center retrospective study (Kelly et al., 2016), only 19.6% of *C. difficile* detection were considered appropriate, with uncertain and inappropriate detection rates of 65.5% and 14.8%, respectively. Therefore, it is necessary to find new biomarkers for differential diagnosis and severity assessments of CDI.

**FIGURE 1:**
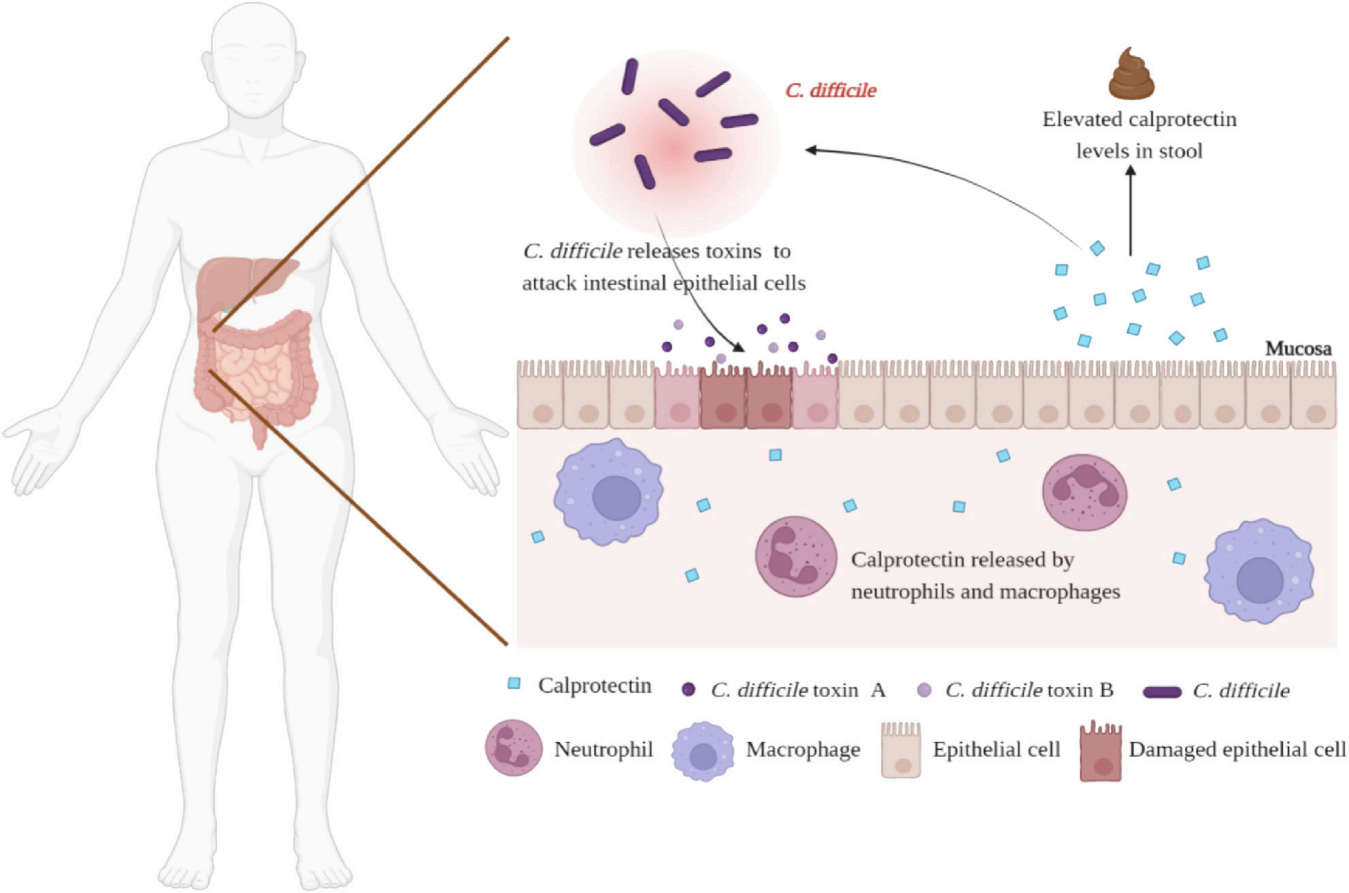
Schematic diagram of elevated fCP levels in CDI patients.

Calprotectin is a 36 kDa member of the S100 protein family, secreted by neutrophils, macrophages, and monocytes. (Khaki-Khatibi et al., 2020). As markers of inflammation, serum and salivary calprotectin have potential applications in a variety of inflammatory diseases, such as arthritis and bacterial infections (Decembrino et al., 2015; Guo et al., 2016; Bartáková et al., 2019). In addition to serum and saliva, calprotectin is also present in feces. Under normal circumstances, the concentration of calprotectin in feces is six times higher than in plasma and is stable at room temperature, giving it an advantage as a biomarker of gastrointestinal inflammation. (Naess-Andresen et al., 1995). The efficacy of fecal calprotectin (fCP) in the diagnosis and prognosis prediction of inflammatory bowel disease (IBD) has been evaluated, including differentiating IBD and irritable bowel syndrome (IBS), predicting disease recurrence and treatment response and evaluating endoscopic activity and disease histological activity (Kalantari et al., 2015; Kalla et al., 2016; Moein et al., 2017; Mak et al., 2018; Reenaers et al., 2018). Notably, CDI can also promote the activation and recruitment of neutrophils and cause inflammation. (Figure 1). Therefore, from this point of view, fCP levels in CDI patients may be elevated and proportional to the degree of intestinal inflammation. In 2008, Shastri et al. (2008) evaluated the role of fCP in the diagnosis of acute diarrhea for the first time and found that patients with CDI had the highest levels of fCP compared with patients with other causes of diarrhea, suggesting that fCP may have value in auxiliary diagnosis of CDI. In recent years, scholars have further explored the characteristics of fCP in CDI patients to examine its potential value. To this end, this review systematically retrieved and summarized relevant studies to comprehensively assess the potential value of fCP in CDI.

## 2 Materials and methods

### 2.1 Definition

CDI is defined as a patient with: (1) presence of diarrhea, defined as 3 or more unformed stools within 1–8 h in 24 or less consecutive hours; (2) positive stool test results in the presence of toxigenic *C. difficile* or its toxins, or colonoscopy or histopathology showing pseudomembranous colitis (Debast et al., 2014). Recurrent CDI (rCDI) was defined as the development of subsequent CDI episodes up to a period of 60 or 90 days following treatment of the initial episode. *C. diffcile* colonization patients was defined as the patients were admitted for at least 72 h, who had received at least 1 dose of an antibiotic within the past 7 days, and did not have diarrhea, on the premise of positive NAAT (Kelly et al., 2020). Treatment response was defined as a decrease in stool frequency or improvement in stool consistency and improvement in disease severity parameters (clinical, laboratory, radiological) after treatment without new signs of severe disease (Debast et al., 2014).

### 2.2 Data sources and search strategy

This systematic review was conducted according to the Preferred Reporting Items for Systematic Reviews and Meta-Analyses (PRISMA) statement guidelines (Moher et al., 2009), using the databases PubMed, Scopus, Ovid, Embase, Cochrane, CNKI, Wanfang, VIP and Siomed. The last search was performed on 6 June 2022, the search formula included “Leukocyte L1 Antigen Complex” and “*Clostridioides difficile*” as medical subject heading (MeSH) terms that were combined in the PubMed advanced search generator. In other databases, combinations of the following keywords were used: “*Clostridium difficile*” or “*Clostridioides difficile*” and “Leukocyte L1 Antigen Complex” or “Calcium-Binding Myeloid Protein P8,14” or “Calcium Binding Myeloid Protein P8,14” or “Calgranulin” or “Calprotectin” or “Migratory Inhibitory Factor-Related Protein MRP” or “Migratory Inhibitory Factor Related Protein MRP” or “Myelomonocytic Antigen L1” or “Antigen L1, Myelomonocytic” or “L1 Antigen” or “Antigen, L1” or “27E10 Antigen” or “Antigen, 27E10” or “Leukocyte L1 Protein” or “L1 Protein, Leukocyte”.

**FIGURE 2:**
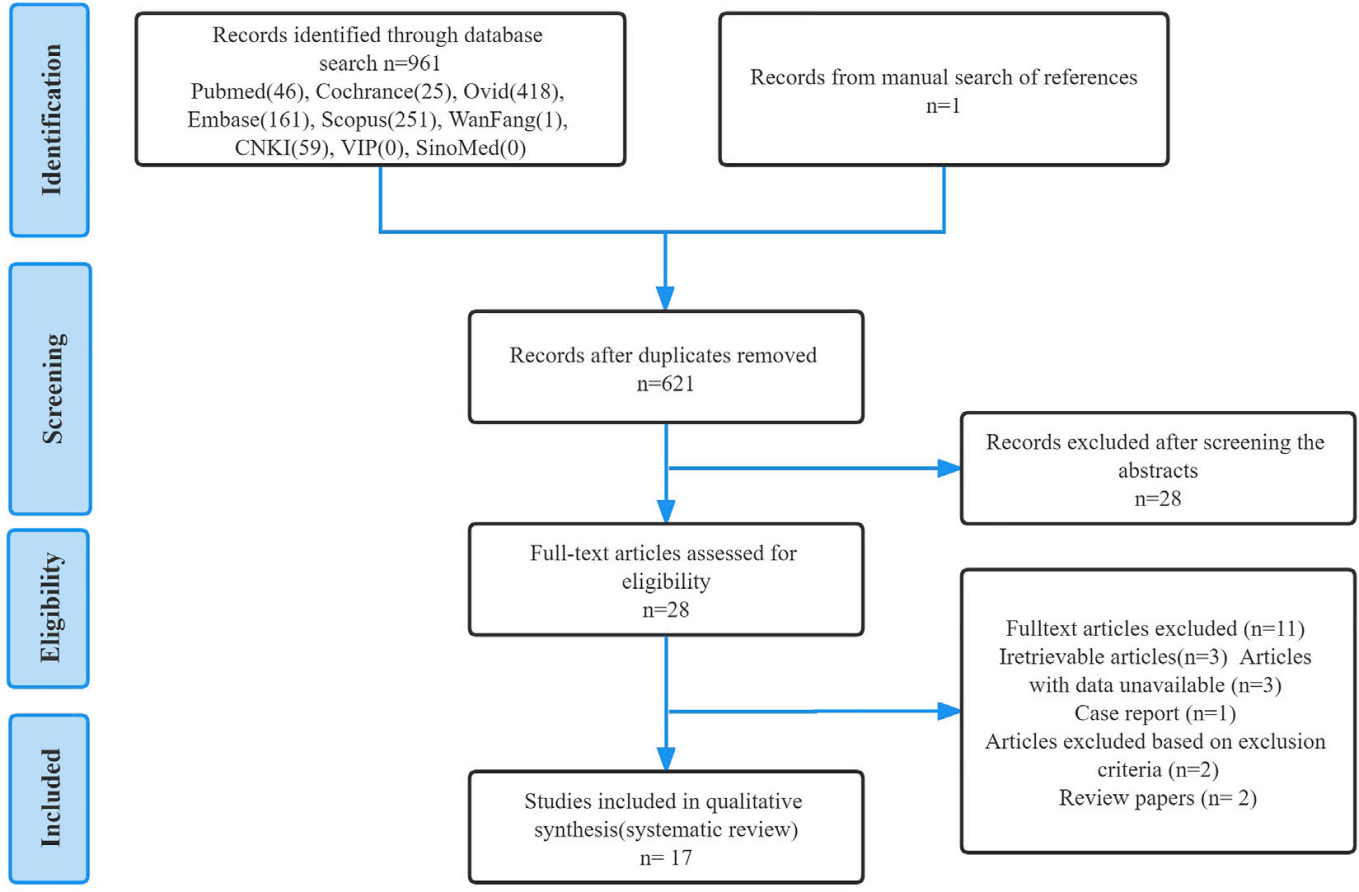
PRISMA flow diagram presenting the detailed search strategy.

Titles and abstracts were independently screened using selection criteria to identify eligible studies. Then, the full text of the study was carefully evaluated and the study was included or excluded accordingly. For any papers with contentious content, consensus discussions were had and agreements were reached to eliminate any ambiguity. Finally, a manual search was performed for any articles in the reference lists of included studies that were missed during the electronic search process. The detailed search flowchart is presented in Figure 2 (in the Results section).

### 2.3 Eligibility criteria and data extraction

Full article studies were included if they met the following criteria: (a) written in English or Chinese, (b) included individuals who were positive for toxigenic *C. difficile* or its toxin or toxin gene and had fCP levels tested, (c) were observational studies, including case-control, cohort and cross-sectional studies. The outcomes of interest included correlations between fCP levels and all CDI-related events (diagnosis, severity assessment, prognosis prediction, etc.) and differences in fCP concentrations in different patient groups. Articles that did not describe clinical symptoms (e.g., diarrhea) in individuals who provided stool samples were excluded. (Table 1).

**TABLE 1:** Eligibility criteria.

PICOS	Inclusion	Exclusion
Participants	Individuals who have undergone both laboratory testing for *C. difficile* and fecal calprotectin testing	Whose clinical symptoms were not described in the article
Intervention	Calprotectin level measured	Other diagnostic parameters used
Comparison	Not applicable	—
Outcome	Difference in calprotectin levels between the groups, association between calprotectin level and all CDI -related events (diagnosis, severity assessment, prognosis prediction, etc.)	—
Study design	Observational clinical studies, case-control, cohort and cross-sectional studies	Opinion papers, review papers, healthcare guidelines, case reports, non-human studies, animal model and *in-vitro* studies

Abbreviations: CDI, Clostridioides difficile infection.

The heterogeneity of CDI-related events as well as that of participants did not allow for a meta-analysis of the studies included in the present systematic review to be performed.

Two study investigators extracted the data independently. Data extraction was conducted for study characteristics (author name, year, study design, comparison groups, topics covered, outcomes measures, fCP detection methods and kits, cutoffs recommended by the kits and whether the CDI meets the definition) (Table 2). In the study by Han et al. (Han et al., 2020) we only extracted data from Group III because only this group included patients with CDI.

**TABLE 2:** Characteristics of included studies.

Study	Design	Comparison groups	Topics covered	Outcomes measures	Comorbidities	fCP detection methods and kits	Cutoffs recommended by the kits	Whether the CDI meets the definition
Aletaha et al. (2019)	Case-control	1) UC + CDI vs. UC	1	*p*-value	UC	Not reported	Not reported	No
Antonella et al. (2020)	Cohort	1) R vs. NR	3	*p*-value	rCDI	ELISA; Calprest; Eurospital Spa, Trieste, Italy	Not reported	Yes
Antonella et al. (2018)	Cohort	1) CDI vs. healthy controls	1	*p*-value	Not reported	ELISA; Calprest; Eurospital Spa, Trieste, Italy	100 µg/g	Yes
		2) Correlation with CSI and SSACG score	2	*p*-value				
		3) R vs. NR	3	*p*-value				
		4) Correlation with ATLAS score	3	*p*-value				
		5) Recurrence vs. no recurrence	3	*p*-value				
		6) CDI-related deaths vs. others	3	*p*-value				
Barbut et al. (2017)	Case-control	1) CDI vs. diarrhea without *C. difficile* vs. diarrhea with non-toxigenic *C. diffcile*	1	*p*-value	Not reported	Lateral flow assay; Quantum Blue, Bühlmann, Basel, Switzerland	Not reported	Yes
		2) Detectable toxins vs. without free toxin	4	*p*-value				
Han et al. (2020)	Cohort	1) CDI vs. diarrhea with non-toxigenic *C. diffcile*	1	*p*-value	Not reported	Fluoroenzyme immunoassay; EliA calprotectin, Thermo Fisher Scientific, Waltham, MA, United States	Not reported	Yes
Hanania et al. (2016)	Case-control	1) Severe CDI vs. non-severe CDI vs. non-CDI AAD	1, 2	*p*-value and AUC	Not reported	ELISA; Eagle Biosciences Inc., Nashua, NH	Not reported	Not reported
Hibbard et al. (2019)	Cohort	1) FMT failure vs. FMT cure	3	*p*-value	All rCDI; 29 IBD	ELISA; Eagle Biosciences, Amherst, NH	Not reported	Yes
Jieun et al. (2017)	Case-control	1) CDI vs. healthy controls	1	*p*-value and AUC	Not reported	ELISA; Bühlmann Laboratories AG, Schönenbuch, Switzerland	Not reported	Yes
		2) Severe CDI vs. non-severe CDI	2	*p*-value and AUC				
Kelly et al. (2020)	Cohort	1) CDI vs. C. diffcile colonization	1	*p*-value	Not reported	ELISA; Bühlmann Laboratories	Not reported	Yes
Rao et al. (2016)	Cohort	1) Correlation with complicated and recurrent CDI	3	*p*-value	Not reported	Lateral flow assay; Quantum Blue, Bühlmann, Basel, Switzerland	Not reported	Yes
		2) TOX+ NAAT- vs. TOX- NAAT+	4	*p*-value				
Mirosław et al. (2019)	Cohort	1) Severe CDI vs. non-severe CDI	2	*p*-value	Not reported	EIA; Ridascreen® Calprotectin immunoassay, R-Biopharm AG	Not reported	Yes
		2) CDI-related deaths vs. others	3	*p*-value				
Nicholson et al. (2017)	Cohort	1) Recurrence vs. no recurrence	3	*p*-value	Not reported	ELISA; Eagle Biosciences, Nashua, NH	Not reported	Yes
Peretz et al. (2016)	Case-control	1) Correlation with Clostridium severity score	2	*p*-value	Not reported	Chemiluminescent immunoassay; Liaison® Calprotectin Saluggia, Italy	Not reported	Not reported
		2) Recurrence vs. no recurrence	3	*p*-value				
Suarez-Carantoña et al. (2021)	Cohort	1) Presumed CDI treated vs. doubtful CDI treated vs. non-treated patients	4	*p*-value and AUC	Not reported	EIA; Calprotectina Blister, Vircell lab, Granada, Spain	Not reported	No
		2) Tox + vs. Tox-/NAAT+	4	*p*-value				
Swale et al. (2017)	Cohort	1) CDI vs. non-CDI AAD	1	*p*-value and AUC	Not reported	ELISA; Calpro, Lysaker, Norway	50 mg/kg	Yes
		2) Severe CDI vs. non-severe CDI	2	*p*-value				
		3) Recurrence vs. no recurrence	3	*p*-value				
		4) CDI-related deaths vs. others	3	*p*-value				
He et al. (2018)	Case-control	1) CDI vs. non-CDI diarrhea	1	*p*-value	Cancer	EIA; Calpro AS, Oslo, Norway	Not reported	Yes
		2) Severe to complicated CDI vs. mild to moderate CDI	2	*p*-value and correlation				
		3) GDH+ TOX+ PCR+ vs. GDH+ TOX-PCR+	4	*p*-value				
Voicu et al. (2021)	Cohort	1) Severe CDI vs. non-severe CDI	2	*p*-value and AUC	Not reported	ELISA	50 μg/g	Yes

Abbreviations: AAD, antibiotic-associated diarrhea; ATLAS, age, treatment with systemic antibiotics, leucocyte count, albumin and serum creatinine; AUC, area under the ROC curve; CDI, *Clostridioides difficile* infection; CSI, CDI severity index; EIA, enzyme immunoassays; ELISA, enzyme-linked immunosorbent assay; fCP, fecal calprotectin; FMT, fecal microbiota transplantation; GDH, glutamate dehydrogenase; IBD, inflammatory bowel disease; NAAT, nucleic acid amplification assays; NR, non-responders; PCR, polymerase chain reaction; rCDI, recurrent CDI; R, responders; SSACG, Scoring System American College of Gastroenterology; TOX, direct toxin test; UC, ulcerative colitis.

### 2.4 Quality assessment

Each of the included studies was independently assessed for quality by two authors using the Newcastle-Ottawa scale (Stang, 2010), and disagreements were resolved by discussion between them. This scale is a validated tool to evaluate the risk of bias in non-randomized studies, including case-control and cohort studies. It comprises three main parameters: selection, comparability and exposure/outcome. The ratings and overall scores of each study are presented in Table 3. Each study was scored as low (<5), medium (5–7) or high (>7) quality.

**TABLE 3:** Quality scores of included studies.

Study	Selection	Comparability	Exposure	Total scores	Study quality
Aletaha et al. (2019)	2	1	2	5	Medium
Antonella et al. (2020)	4	2	2	8	High
Antonella et al. (2018)	3	1	3	7	Medium
Barbut et al. (2017)	3	1	2	6	Medium
Han et al. (2020)	4	1	2	7	Medium
Hanania et al. (2016)	3	1	1	5	Medium
Hibbard et al. (2019)	4	1	2	7	Medium
Jieun et al. (2017)	3	2	2	7	Medium
Kelly et al. (2020)	4	2	1	7	Medium
Rao et al. (2016)	2	1	2	5	Medium
Mirosław et al. (2019)	4	2	2	8	High
Nicholson et al. (2017)	2	2	2	6	Medium
Peretz et al. (2016)	2	1	2	5	Medium
Suarez-Carantoña et al. (2021)	3	1	2	6	Medium
Swale et al. (2017)	3	2	2	7	Medium
He et al. (2018)	2	0	2	4	Low
Voicu et al. (2021)	3	2	2	7	Medium

## 3 Results

### 3.1 Search results

As outlined in Figure 2, a total of 962 studies were identified through database and manual searches, of which 934 studies were excluded after title and abstract screening. The remaining 28 studies were further assessed for eligibility by reading the full text. Finally, 17 studies met all inclusion criteria and were included in the systematic review.

### 3.2 Characteristics and quality of included studies

Table 2 summarizes the characteristics of the included studies. Of the 17 studies, 11 were cohort studies and 6 were case-control studies. Based on the content of these studies, we divided them into 4 topics. Topic 1: fCP in differentiating patients with CDI from other populations. Topic 2: fCP in assessing the severity of CDI. Topic 3: fCP in predicting the prognosis of CDI patients. Topic 4: fCP in other aspects of CDI. Overall, 9 studies focused on topic 1, 8 studies focused on topic 2, 9 studies included topic 3, and 4 studies addressed topic 4. All studies used EIA to measure fCP levels, except for one study that was not reported. Additionally, patients with CDI in 13 studies met our defined criteria. Based on quality scores, two studies were considered high quality, one study was low quality, and the remaining studies were identified as medium quality (Table 3).

### 3.3 Fecal calprotectin in differentiating patients with *Clostridioides difficile* infection from other populations

Nine studies assessed differences in fCP concentrations between patients with CDI and other populations (Table 4).

**TABLE 4:** Main results of fCP in distinguishing patients with CDI from other populations.

Study	Comparison groups	fCP level (µg/g)	Positivity	Associated outcomes	
				*p*-Value	AUC
CDI vs. healthy controls					
Antonella et al. (2018)	56 CDI	354 ± 216	—	< 0.001	
	50 healthy controls	29 ± 21	—		
Jieun et al. (2017)	I: 30 severe CDI	1391.5 (170.0–2088.1)	—	I vs. II: < 0.001	0.82 (CDI vs. healthy controls)
	II: 50 mild CDI	188.2 (41.4–188.2)	—	II vs. III: 0.019	
	III: 71 healthy controls	35.6 (10.7–108.9)	—	I vs. III: < 0.001	
UC with CDI vs. UC without CDI					
Aletaha et al. (2019)	35 UC with CDI	—	94.30%	0.001	
	31 UC without CDI	—	56.70%		
CDI vs. non-CDI diarrhea					
Barbut et al. (2017)	I: 135 CDI	218.0 (67.2–795.5)	—	I vs. II: 0.001	
	II: 135 diarrhea without *C. difficile*	111.5 (34.8–374.5)	—		
	III: 50 diarrhea with non-toxigenic *C. diffcile*	111.3 (43.9–374.8)	—	I vs. III: 0.011	
Han et al. (2020)	69 CDI	—	—	0.273	
	20 diarrhea with non-toxigenic *C. diffcile*	—	—		
Hanania et al. (2016)	I:50 severe CDI	276 (15–6275)			0.70 (CDI vs. non-CDI AAD)
	II:50 non-severe CDI	11 (0–1261)			
	III:50 non-CDI AAD	16 (0–293)			
Swale et al. (2017)	159 CDI	684.8 (203.7–1,581.0)		< 0.0001	0.86
	51 non-CDI AAD	66.5 (23.1–145.7)			
He et al. (2018)	117 CDI	183.6	—	0.006	
	115 non-CDI diarrhea	145.6	—		
CDI vs. *C. diffcile* colonization					
Kelly et al. (2020)	120 CDI	290.8 (64.6–888.3)		0.088	
	43 *C. diffcile* colonization	174.9 (75.3–409.2)			

Abbreviations: AAD, antibiotic-associated diarrhea; AUC, area under the ROC, curve; CDI, Clostridioides difficile infection; fCP, fecal calprotectin; UC, ulcerative colitis.

#### 3.3.1 *Clostridioides difficile* infection vs. healthy controls

Two studies showed that patients with CDI had significantly higher fCP levels than healthy subjects. Receiver operating characteristics (ROC) curves showed that the best functional connectivity (FC) value for distinguishing between CDI and healthy subjects was 112.5 μg/g, the area under the curve (AUC) was 0.821, sensitivity was 75% and specificity was 79%.

#### 3.3.2 Ulcerative colitis with *Clostridioides difficile* infection vs. Ulcerative colitis without *Clostridioides difficile* infection

Aletaha et al. (Aletaha et al., 2019) found that UC patients with CDI had a higher rate of fCP positivity compared with UC patients without CDI, but this study did not report a threshold for fCP positivity.

#### 3.3.3 *Clostridioides difficile* infection vs. *C. diffcile* colonization

One study (Kelly et al., 2020) observed higher fCP levels in CDI patients than in patients with asymptomatic colonization with *C. difficile*, but this difference was not statistically significant.

#### 3.3.4 *Clostridioides difficile* infection vs. non-*Clostridioides difficile* infection diarrhea

Three studies observed higher fCP levels in patients with CDI compared to patients with diarrhea from other causes (Barbut et al., 2017; Swale et al., 2017; He et al., 2018). Another study (Han et al., 2020) did not observe a significant difference between the two. In addition, two studies conducted ROC analysis on the ability of fCP to distinguish CDI patients from patients with non-CDI AAD and found AUC values of 0.70 and 0.86, respectively (Hanania et al., 2016; Swale et al., 2017).

### 3.4 Fecal calprotectin in assessing the severity of *Clostridioides difficile* infection

As shown in the Table 5, there are eight studies exploring the feasibility of using fCP to assess the severity of CDI. The study of Jieun et al. (2017) showed that the area under the ROC curves were 0.821 and 0.746 with a sensitivity of 75% and 70% and specificity of 79% and 80%, for severe versus mild cases, respectively. Another study yielded sensitivity and specificity of fCP for distinguishing severe CDI from non-severe CDI and non-CDI AAD are 57% and 88% (Hanania et al., 2016). Voicu et al. (2021) suggested a cut-off of 290.09 μg/g for the predictive marker of fCP, which permitted to identify patients with severe and mild CDI, having 100% sensitivity and 76% specificity. Only two studies showed no correlation between fCP levels and patients’ clinical scores. However, one of them found a trend for higher fCP levels in patients with a higher *Clostridium* severity score index (*p* = 0.0633). Other studies show higher fCP levels in severe CDI patients compared to non-severe CDI patients, although one of these studies did not reach statistical significance.

**TABLE 5:** Main results of studies that explored the relationship between fCP and the severity of CDI.

Study	Comparison groups	Results (µg/g)	Associated outcomes	
			*p*-Value	AUC
Antonella et al. (2018)	56 CDI	Correlation with CSI and SSACG score	both >0.05	
Hanania et al. (2016)	50 severe CDI	276 (15–6275)		0.84 (severe CDI vs. non-severe CDI and non-CDI AAD)
	50 non-severe CDI	11 (0–1261)		
	50 non-CDI AAD	16 (0–293)		
Jieun et al. (2017)	30 severe CDI	1391.5 (173.5–2075.9)	< 0.001	0.746
	50 non-severe CDI	188.2 (41.4–591.6)		
Mirosław et al. (2019)	50 severe CDI	770 (689–802)	0.009	
	26 non-severe CDI	659 (369–775)		
	31 severe CDI	780 (714–810)	0.001	
	45 non-severe CDI	661 (581–789)		
Peretz et al. (2016)	29 CDI	Correlation with Clostridium severity score index	0.0633	
Swale et al. (2017)	47 severe CDI	969.3	0.09	
	112 non-severe CDI	512.7		
He et al. (2018)	22 severe to complicated CDI	218.5	0.014	
	95 mild to moderate CDI	182.1		
Voicu et al. (2021)	18 severe CDI	615.14 (403.62–784.4)	<0.001	0.953
	41 non-severe CDI	195.42 (131.12–298.59)		

Abbreviations: AAD, antibiotic-associated diarrhea; AUC, area under the ROC, curve; CDI, Clostridioides difficile infection; CSI, CDI, severity index; fCP, fecal calprotectin; SSACG, Scoring System American College of Gastroenterology.

### 3.5 Fecal calprotectin in predicting the prognosis of *Clostridioides difficile* infection patients

Eight studies assessed the prognostic value (response to therapy, disease recurrence, death, etc.) of fCP in patients with CDI, as shown in Table 6.

**TABLE 6:** Main results of studies that explored the prognostic value of fCP in patients with CDI.

Study	Comparison groups	Results (µg/g)	Associated outcomes
Respond to therapy			
Antonella et al. (2020)	13 R vs. 15 NR	T0:298.8 (230–450) vs. 620 (354.7–2392.2)	*p* = 0.07
		T1:464.8 (244.6–929.4) vs. 483.8 (254.5–3085)	*p* = 0.75
		T2:320 (175.5–713.3) vs. 440 (223.1–757.2)	*p* = 0.61
Antonella et al. (2018)	33 R vs. 23 NR	320 ± 201 vs. 439 ± 267	*p* > 0.05
		Correlation with ATLAS score	*p* > 0.05
CDI recurrence			
Antonella et al. (2018)	Recurrence vs. no recurrence	444 ± 163 vs. 329 ± 230	*p* > 0.05
Hibbard et al. (2019)	11 FMT failure vs. 123 FMT cure	T0: 84.3 (35.7–9089.9) vs. 43.6 (32.4–11430.9)	*p* = 0.0848
		T1: 450.0 (35.0–10733.2) vs. 46.3 (0–4296.9)	*p* = 0.0183
Nicholson et al. (2017)	8 Recurrence vs. 19 no recurrence	14.4 (8.7–121.9) vs. 8.6 (8.1–18.1)	*p* = 0.38
Peretz et al. (2016)	Recurrent CDI vs. Non-recurrence CDI	284.7 (46–840) vs. 356.1 (21–932)	*p* = 0.662
Swale et al. (2017)	50 Recurrence vs. 62 no recurrence	—	*p* = 0.53
CDI related deaths			
Antonella et al. (2018)	CDI-related deaths vs. others	384 ± 195 vs. 345 ± 224	*p* > 0.05
Mirosław et al. (2019)	13 CDI-related deaths vs. 63 others	772 (693–800) vs. 727 (607–798)	*p* = 0.27
Swale et al. (2017)	14 CDI-related deaths vs. 145 others	—	*p* = 0.5
Other outcomes			
Rao et al. (2016)		Correlation with complicated and recurrent CDI	(OR 24.9, 95% CI 2.4–257.9, *p* = 0.007)

Abbreviations: ATLAS, age, treatment with systemic antibiotics, leucocyte count, albumin and serum creatinine; CDI, Clostridioides difficile infection; fCP, fecal calprotectin; NR, non-responders; R, responders; T0, before treatment; T1, T2, after treatment.

#### 3.5.1 Response to therapy

There are two studies compared concentrations of fCP between patients who did and did not respond to treatment. The results showed no significant difference in fCP levels between responders and non-responders, either before or after treatment, although responders had lower fCP levels. Additionally, the study by Antonella et al. (2018) showed no correlation between fCP levels and ATLAS scores, which assess treatment response.

#### 3.5.2 *Clostridioides difficile* infection recurrence

Five studies compared fCP levels in rCDI patients with those without recurrence. Only one study showed significantly higher fCP levels in patients with rCDI after fecal microbiota transplantation (FMT) than in patients without recurrence, and results from other studies showed no statistically significant difference between the two groups of patients.

#### 3.5.3 *Clostridioides difficile* infection related death

In all three studies, there were no statistically significant differences in fCP levels in CDI-related deaths compared with surviving subjects.

#### 3.5.4 Other outcomes

Rao et al. (2016) found that patients with complicated/recurrent CDI (adverse outcomes) have higher normalized fCP levels. Further modeled as a diagnostic test, a high normalized fCP was 38.5% sensitive and 91.9% specific for complicated/recurrent CDI, suggesting that high fCP levels were associated with adverse outcomes in CDI.

### 3.6 Fecal calprotectin in other aspects of *Clostridioides difficile* infection

Four studies compared fCP levels in stool samples that were positive for direct toxin testing and those with no detectable toxin. All of these results showed higher levels of fCP in samples positive for direct toxin assays, even though two of the studies did not reach statistical differences (Table 7).

**TABLE 7:** Main results of fCP in other aspects of CDI.

Study	Comparison groups	Results (µg/g)	*p*-Value	AUC
Barbut et al. (2017)	87 detectable toxins vs. 48 without free toxin	274.0 (85.8–1321.0) vs. 166.0 (47.0–535.0)	0.051	
Rao et al. (2016)	20 TOX+ NAAT- vs. 30 TOX- NAAT+	—	> 0.05	
Suarez-Carantoña et al. (2021)	TOX+ vs. TOX−/NAAT+	—	< 0.05	
He et al. (2018)	24 GDH+/TOX+/PCR + vs. 86 GDH+/TOX−/PCR+	200.2 vs. 182.8	0.044	
Management				
Suarez-Carantoña et al. (2021)	83 presumed CDI treated vs. 25 doubtful CDI treated vs. 26 non-treated patients	410 (138–815) vs. 188 (57–524) vs. 51 (26–97)	< 0.001	0.884

Abbreviations: AUC, area under the ROC, curve; GDH, glutamate dehydrogenase; NAAT, nucleic acid amplification assays; PCR, polymerase chain reaction; TOX, direct toxin test.

Suarez-Carantoña et al. (2021) divided patients into three groups: group I, recommended treatment for hypothetical CDI; group II, uncertain diagnosis but patients treated for CDI; and group III, assumed *C. difficile* colonization or self-limiting CDI that did not require treatment according to the recommendations of clinicians and professional consultants. After comparing the fCP levels of the three groups of patients, it was found that the fCP levels of the patients in group I were significantly higher than those in the other two groups. At the same time, the fCP level of patients in group II was significantly higher than that in group III (Table 7).

## 4 Discussion

Currently, there has been a lack of suitable biomarkers for the diagnosis, disease severity assessment and prognosis prediction of CDI patients. In recent years, several studies have explored the potential application value of fCP in CDI. However, there is conflict and controversy among their results. This review comprehensively summarizes the relevant studies in this field. Overall, a certain degree of inconsistency among study results was observed across topics. Nonetheless, we analyze the results and present our own insights based on their study design and methodology.

### 4.1 The low application value of fecal calprotectin in the diagnosis of *Clostridioides difficile* infection

To date, research on fCP in the diagnosis of CDI has mainly focused on four issues. The first is whether fCP can distinguish CDI patients from healthy controls. Studies by Antonella et al. (2018) and Jieun et al. (2017) both showed that levels of fCP in CDI patients were significantly higher than those in healthy subjects. Average fCP levels of healthy controls in the two studies were below 50 μg/g, which was consistent with previous fCP data reported in other studies. The ROC curve showed good discriminative ability of fCP for CDI patients and healthy subjects, indicating that fCP has the basic condition as an inflammatory marker. However, it may not be of much help to clinicians because most of the time the problem is to distinguish patients with CDI from those with diarrhea from other causes, rather than healthy individuals. The second issue is whether fCP can distinguish CDI patients from IBD patients. Only one study focused on this issue and found that the positivity rate of fCP in UC patients with positive CDI test was significantly higher than that in patients with negative CDI test. It should be noted that even in UC patients with negative CDI test, the fCP positive rate reached 56%, and the cut-off value of fCP was not mentioned in the study. Therefore, further studies are needed to assess the ability of fCP to differentiate CDI from IBD patients. The third issue is whether fCP can distinguish CDI patients from non-CDI patients with diarrhea. Three of the four studies observed significantly higher levels of fCP in CDI patients compared with non-CDI diarrhea patients. Two studies conducted ROC analysis on the ability of fCP to distinguish CDI patients from non-CDI AAD patients and found AUC values of 0.70 and 0.86, respectively. These results suggest that fCP has some utility in distinguishing CDI patients from non-CDI diarrhea patients, and may be useful for screening patients with diarrhea for CDI, but would not add much value to the currently available diagnostic paradigm. The fourth issue is whether fCP can distinguish CDI patients from those colonized by *C. difficile*. One study evaluated differences in fCP levels between CDI and toxigenic *C. difficile*-colonized patients, and no significance was observed. To date, we have not found any studies evaluating the ability of fCP to differentiate *C. difficile* infection from colonization by ROC curve.

Judging from the current data, although the level of fCP in CDI patients is higher than that in other populations, and fCP has shown good discriminative ability in some studies, its value for improving current CDI diagnosis methods may be very limited. On one hand, we observed that fCP levels vary widely, and there was significant overlap between CDI patients and control groups, making it difficult to determine optimal cut-off values for fCP and reducing the accuracy of CDI predictions. Even though part of the reason for the large inter-individual variability may be due to differences in the kits and methods used to detect fCP. On the other hand, other intestinal inflammatory diseases can also lead to elevated fCP (Kopylov et al., 2014), which is especially important for CDI because infected patients are usually elderly and accompanied by multiple comorbidities. Nevertheless, a study by Whitehead et al. (2014) reported a sensitivity of 96% for fCP >50 mg/g to discriminate *C. difficile*-positive samples in stool samples from a cohort of patients with diarrhea. Therefore, fCP may have some value for screening CDI patients with diarrhea. Finally, further studies are needed to evaluate the ability of fCP to distinguish CDI patients from those with asymptomatic colonization and IBD.

### 4.2 The relationship between fecal calprotectin levels and the severity of *Clostridioides difficile* infection

According to guideline recommendations (Van Prehn et al., 2021), there are different treatment options for CDI patients of different severity. Therefore, it is important to use reliable biomarkers to confirm the severity of infections. However, current diagnostic methods for CDI are still unable to determine the severity of CDI. Clinicians make condition assessments mainly on the clinical manifestations and risk factors of patients. To distinguish mild from severe CDI, the 2010 Society for Healthcare Epidemiology of America and Infectious Diseases Society of America Clinical Practice Guidelines (Cohen et al., 2010) and the European Society of Clinical Microbiology and Infectious Diseases guidelines define criteria based on patient age, physical signs and complications as well as serum albumin, creatinine and leukocytes. Neutrophils, a major leukocyte, play an important role in the pathogenesis of CDI, and fCP secreted by neutrophils is considered by some to be a potential biomarker of disease activity.

In this review, 5 relevant studies all showed higher fCP levels in patients with severe CDI, although a statistical difference was not reached in one of the studies. Moreover, based on the AUC values, sensitivity, and specificity reported in 3 studies, fCP showed a relatively strong ability to distinguish patients with severe CDI from patients with non-severe CDI or non-CDI AAD. However, we also found inconsistencies in their results. For example, the median fCP in patients with severe CDI in different studies ranged from a minimum of 218.5 µg/g to a maximum of 1391.5 µg/g. This discrepancy can be attributed in part to differences in fCP detection kits and in part to differences in the criteria for assessing the severity of CDI. In addition, two studies failed to observe a correlation between fCP levels and three index scores reflecting the severity of CDI. Overall, although the criteria for assessing the severity of CDI differed in different studies, most studies supported the potential value of fCP for assessing disease severity. Current studies have found that fCP levels are significantly related to higher peripheral blood white blood cell counts, and the higher the intensity of CDI inflammation, the greater the increase in neutrophil counts, which may reflect the relationship between fCP levels and the degree of intestinal inflammation. Therefore, from this point of view, fCP may play a role in assessing the severity of CDI. However, it should be noted that large variability in observed fCP levels may also complicate the formulation of optimal cut-off values for severe and non-severe CDI. Therefore, more prospectively designed studies with large sample sizes are needed to further evaluate the ability of fCP to differentiate patients with severe CDI. In addition, prior to this, it is important to unify the criteria for defining severe CDI, as this will improve comparability between different studies.

### 4.3 Single fecal calprotectin may not predict prognosis in patients with *Clostridioides difficile* infection

So far, the clinical scoring system that has been proposed to predict the prognosis of CDI is mainly based on a combination of clinical, laboratory and radiology/endoscopic parameters (Belmares et al., 2007; Fujitani et al., 2011; Miller et al., 2013). However, colonoscopy and abdominal CT examination are neither commonly performed on these patients nor are they easily obtained, so the effectiveness of these scores in clinical practice is still limited. Here we describe several studies that have evaluated the prognostic value of fCP in CDI.

Two studies evaluated the value of fCP in predicting patient response to treatment. One study classified patients into “responders” and “non-responders” based on the presence or absence of diarrhea relief and improvement in clinical picture, and assessed treatment response by ATLAS scores based on patient age, antibiotic treatment, white blood cell count, albumin, and serum creatinine. Another study classified patients as “responders” and “non-responder” based on whether they had diarrhea at 8 weeks. Neither study observed a difference in fCP levels between “responders” and “non-responders” or a correlation between treatment response and fCP levels, even when patients received different treatment options. The level of fCP may be related to the patient’s disease state at the time of stool collection, as levels of fCP may be higher during an acute CDI episode. In the study of Antonella et al. (2020), they measured the fCP levels of patients before treatment (T0) and after treatment (T1, T2). However, no differences were observed between responders and non-responders. One possibility is that the clinical response could be related to the difference in fCP levels before and after treatment. Perhaps it is more appropriate to evaluate patients’ responses to treatment in conjunction with the degree of reduction in fCP after treatment.

Based on current data, there is insufficient evidence that fCP levels at the time of CDI diagnosis predict disease recurrence and related death. In the study by Hibbard et al. (2019), there was no significant difference in fCP levels before FMT between FMT-cured (no episodes of CDI during the 60 days after FMT) and FMT-failure patients. Relatively speaking, fCP levels on day 7 after FMT were more valuable in predicting response to FMT. At the same time, one study showed that elevated fCP is a risk factor for patients with adverse outcomes (complexity and recurrence of CDI). We do not believe that fCP levels at the time of diagnosis are suitable for predicting patient outcomes because the time span between the measurement of fCP and the appearance of adverse outcomes is too long. Patients should be followed for a longer period of time and their fCP levels should be continuously measured to better evaluate the value and potential of fCP in the prognosis prediction of CDI. We look forward to more rigorously designed studies with larger sample sizes evaluating this in the future.

### 4.4 Higher fecal calprotectin levels may indicate detectable toxins in stool and a need for treatment in the patient

The use of NAAT in the diagnosis of CDI has resulted in a significant increase in the documented incidence of CDI due to its higher sensitivity. Data have shown that individuals who test positive for both NAAT and direct toxin assays have longer duration of symptoms and hospitalization, as well as higher mortality, than individuals who test positive for NAAT alone. Meanwhile, the duration of symptoms and mortality in NAAT-positive/toxin-negative patients were similar to those in both NAAT- and toxin-negative patients. Therefore, some scholars have questioned the clinical significance of only NAAT positive. Considerable debate remains about how to interpret and manage NAAT-positive/toxin-negative patients. As per the European guidelines for patients with evidence of *C. difficile* but negative toxin test results, patients need to be evaluated clinically as they may have undetectable toxin levels or false negative toxin results or may be potential carriers of toxigenic *C. difficile* (Crobach et al., 2016). Hogan et al. (2022) observed similar clinical outcomes in treated and untreated *C. difficile* NAAT-positive/toxin-negative adult hospitalized patients. These data support the view that a positive direct toxin test is more closely related to infection than a positive toxin gene test. Four studies in this review showed higher levels of fCP in samples positive for direct toxin assays than in samples positive for toxin genes alone or in which toxin was not directly detectable, although no statistical difference was observed in two of them. From this point of view, a high fCP level may indicate a positive direct toxin test and an infection in the patient. In addition, even a positive toxin test result does not always mean a patient’s need for treatment. Suarez-Carantoña et al. (2021) divided patients into those with hypothetical CDI for whom treatment was recommended, those with indeterminate diagnosis but received CDI therapy, and those with *C. difficile* colonization or self-limiting CDI who did not require treatment. After comparing levels of fCP in the three groups of patients, it was found that fCP levels were significantly higher in patients who required treatment than in those who did not. Therefore, fCP should be investigated as a potentially useful marker to indicate whether patients with toxin-producing *C. difficile* require treatment.

### 4.5 Limitations and recommendations for future studies

Several limitations were observed in this systematic review. Firstly, the guidelines and standards (including CDI diagnosis, severity assessment and prognosis assessment) used in various studies were inconsistent, which was a major source of heterogeneity. Secondly, the selection of subjects in some studies were not rigorous enough, which may lead to the inclusion of patients with other underlying diseases that affect the level of fCP. In addition, there were differences in the outcome measures chosen in the studies. Due to this heterogeneity, a meta-analysis was difficult to conduct, and we could not determine calprotectin cut-off values for distinguishing between patients.

Therefore, more high-quality studies are needed to further explore the value of fCP in CDI. Here, we offer some suggestions. First of all, the diagnosis and severity assessment of CDI should strictly follow the criteria prescribed by the guidelines. Second, it is better if the selected control group is matched with the experimental group in terms of age, gender, underlying diseases, etc. Third, we encourage future studies to use ROC curves to assess the discriminative power of fCP in different patients. Finally, multicenter studies with large sample sizes may provide more reliable and convincing results.

## 5 Conclusions

Overall, although the current studies on fCP in CDI are small and preliminary, we still obtained some valuable information. We observed a trend towards higher fCP levels in patients with CDI compared to healthy individuals and patients with diarrhea of other causes. Maybe it can be used for CDI screening but its application value in CDI diagnosis may be low. The potential role of fCP in the assessment of CDI severity warrants further evaluation. In addition, high levels of fCP may indicate the need for treatment. Unfortunately, there is insufficient evidence to suggest that fCP has a prognostic value in CDI. The results analyzed in this systematic review should be interpreted with caution because of differences between study results. Meanwhile, more high-quality studies are needed to further comprehensively evaluate the application value and potential of fCP in CDI.

## Data Availability

The original contributions presented in the study are included in the article/supplementary material, further inquiries can be directed to the corresponding author.
